# Atrial Fibrillation in Adult Congenital Heart Increase Ischemic Stroke Risk Even at Low CHA_2_DS_2_-VASc Score

**DOI:** 10.31083/j.rcm2408225

**Published:** 2023-08-08

**Authors:** Yu-Sheng Lin, Yi-Chun Huang, Chia-Pin Lin, Victor Chien-Chia Wu, Yi-Wei Kao, Hou-Yu Chiang, Pao-Hsien Chu

**Affiliations:** ^1^Division of Cardiology, Chang Gung Memorial Hospital Linkou Medical Center, 333423 Taoyuan, Taiwan; ^2^Healthcare Center, Taoyuan Chang Gung Memorial Hospital, 333008 Taoyuan, Taiwan; ^3^Department of Internal Medicine, Taoyuan Chang Gung Memorial Hospital, 333008 Taoyuan, Taiwan; ^4^Department of Applied Statistics and Information Science, Ming Chuan University, 333321 Taoyuan, Taiwan; ^5^Artificial Intelligence Development Center, Fu Jen Catholic University, 242062 New Taipei City, Taiwan; ^6^Department of anatomy, College of Medicine, Chang Gung University, 333323 Taoyuan, Taiwan; ^7^Graduate Institute of Biomedical Sciences, Chang Gung University, 333323 Taoyuan, Taiwan; ^8^Heart Failure Center, Chang Gung Memorial Hospital Linkou Medical Center, 333423 Taoyuan, Taiwan

**Keywords:** adult congenital heart disease, atrial fibrillation, ischemic stroke, anticoagulation, CHA_2_DS_2_-VASc score

## Abstract

**Background:**

The population of adults with congenital heart diseases (ACHDs) is expanding, and atrial fibrillation (AF) emerges as a crucial risk factor for ischemic stroke. However, the evidence regarding the impact of AF on the incidence of ischemic stroke in ACHDs remains limited. In this study, we aimed to investigate the prevalence and effect of AF among ACHDs and assess the suitability of the traditional CHA₂DS₂-VASc score in this specific population.

**Methods:**

Data of ACHDs from 2000 to 2010 were retrospectively collected from the Taiwan National Health Insurance Research Database. We divided ACHDs into those with and without AF, and ischemic stroke incidence was studied among ACHD subtypes and those who received anticoagulant therapy with warfarin or not according to CHA₂DS₂-VASc score.

**Results:**

36,530 ACHDs were retrieved from the database. ACHDs had a 4.7–15.3 times higher AF risk than did the general population, which varied based on the age group. ACHDs with AF had 1.45 times higher ischemic stroke risk than those without AF (*p* = 0.009). Ischemic stroke incidence among ACHDs with AF aged <50 years was 1.46 times higher than those without AF (*p* = 0.207). Ischemic stroke incidence was over 1.47% even in those with a low CHA₂DS₂-VASc score (0–1) with or without anticoagulant therapy.

**Conclusions:**

During the 12-year follow-up, ACHDs with AF were found to have an increased risk of ischemic stroke. The ischemic stroke incidence was high, even in those with a low CHA₂DS₂-VASc score (0–1).

## 1. Introduction

The prevalence of congenital heart disease (CHD) worldwide is currently estimated to be 9 per 1000 newborns, with significant geographic variability [1, 2, 3, 4, 5]. Although the prevalence of severe CHD is decreasing in many Western/developed countries due to fetal screening and pregnancy termination, the overall global prevalence of CHD is increasing [6]. This may be attributed to various factors such as improved diagnosis and increased survival rates due to advancements in medical, surgical, and technological interventions. In fact, more than 90% of individuals born with CHD now survive into adulthood as a result of these advancements over the past few decades [2, 7, 8]. CHD cannot be completely cured; therefore, adults with CHD (ACHD) have a high risk of cardiovascular complications, including arrhythmia, stroke, heart failure, and myocardial infarction, as well as their early manifestation and a shortened lifespan [9, 10, 11, 12].

Arrhythmia is the most common cause of unscheduled hospital visits in ACHDs and accounts for one-third of all emergency admissions in this population [10, 13]. Atrial fibrillation (AF) is the most powerful risk factor for stroke, conferring a four- to seven-fold increased risk in the general population [14]. Dr. Pedersen [15] used Danish nationwide registries to demonstrate that young ACHD patients have a higher risk of ischemic stroke compared to the general population, even at low ${\mathrm{CHA}}_{2}$${\mathrm{DS}}_{2}$-VASc scores. This finding conflicts with the ${\mathrm{CHA}}_{2}$${\mathrm{DS}}_{2}$-VASc score implication. However, it is important to note that the Pedersen data did not confirm the diagnosis of atrial fibrillation, and further studies are needed to investigate this association [15]. Traditionally, for patients with AF, the ${\mathrm{CHA}}_{2}$${\mathrm{DS}}_{2}$-VASc (congestive heart failure, hypertension, age ≥75 years, diabetes mellitus, stroke or transient ischemic attack, vascular disease, age 65–74 years, sex category) score is used to determine stroke risk and predict anticoagulation [16]. However, ACHDs often do not have typical thromboembolic risk factors [16]. Therefore, the Pediatric and Congenital Electrophysiology Society and Heart Rhythm Society guidelines recommend anticoagulation therapy according to disease complexity and the ${\mathrm{CHA}}_{2}$${\mathrm{DS}}_{2}$-VASc score in ACHDs based on expert consensus [17, 18].

Therefore, this retrospective study evaluated the association between AF and stroke in ACHDs and the accuracy with which the ${\mathrm{CHA}}_{2}$${\mathrm{DS}}_{2}$-VASc score can indicate warfarin use in this population.

## 2. Methods

### 2.1 Data Source

We retrospectively collected the longitudinal claims data of all individuals with CHD between 2000 and 2010 from the Taiwan National Health Insurance Research Database (NHIRD) (http://nhird.nhri.org.tw/date_01.html). The national health insurance program in Taiwan was launched in 1995, and it universally and successfully provides quality health care at an affordable cost. More than 99.6% of the residents of Taiwan are covered under the program, and medical records are stored in the NHIRD, which is updated biannually. All patients with major diseases, including ACHD, must be registered in the Registry for Catastrophic Illness Patients database (http://nhird.nhri.org.tw/date_01.html).

### 2.2 Ascertainment of ACHD, AF, Ischemic Stroke and Comorbidities

In Taiwan, suspected ACHDs are referred to cardiologists for echocardiographic diagnosis and treatment, and the majority of them are followed up at medical centers. The diagnosis of ACHDs, whether inpatient or outpatient, is made based on the International Classification of Diseases, Ninth Revision, Clinical Modification (ICD-9-CM) codes, and the accuracy of the diagnosis is verified by a hospital-based insurance claims data to ensure its validity. Hospitals that file inaccurate claims may be penalized by the National Health Insurance Bureau. In addition, patients who receive a catastrophic illness certificate are exempted from copayments pertaining to their condition in Taiwan. The CHD diagnoses can be classified into cyanotic CHD (tetralogy of Fallot (TOF), ICD-9-CM code 745.2; common truncus, ICD-9-CM code 745.0; double-outlet right ventricle, ICD-9-CM code 745.11; or other cyanotic CHDs, ICD-9-CM codes 745.1, 745.12, 745.3, 746.1, 746.7, and 747.41) and noncyanotic CHD (ventricular septal defect (VSD), ICD-9-CM code 745.4; ostium- or secundum-type atrial septal defect (ASD), ICD-9-CM code 745.5; congenital pulmonary stenosis (PS), ICD-9-CM code 746.02; or other noncyanotic CHDs, ICD-9-CM codes 745.60, 745.6, 746.2, 746.3, 746.82, and 747.1). In addition, patent ductus arteriosus was not included because it is generally not considered to be ACHD.

AF diagnosis was ascertained if an ICD-9-CM code of 427.31 was listed in the secondary discharge diagnosis of stroke hospitalization, in at least one subsequent inpatient claim, or in at least two subsequent outpatient claims. The primary outcome of this study was hospitalization, with a principal discharge diagnosis of stroke events during the study period. The stroke refers to ischemic stroke only (ICD-9-CM codes 433-434), excluding hemorrhagic stroke, cryptogenic stroke and transient ischemic attack (TIA). The diagnostic codes of AF and strokes have been validated in previous NHIRD studies [19, 20, 21, 22, 23].

The comorbidities included hypertension, diabetes mellitus, obstructive sleep apnea, hypothyroidism, congestive heart failure, prior stroke or TIA or thromboembolism, vascular disease, Charlson Comorbidity Index score, and ${\mathrm{CHA}}_{2}$${\mathrm{DS}}_{2}$-VASc score. The presence of each comorbidity was defined as having at least two outpatient diagnoses or anyone inpatient diagnosis in the previous year. However, there was one exception for the previous stroke as one component of ${\mathrm{CHA}}_{2}$${\mathrm{DS}}_{2}$-VASc score. Previous stroke was defined as anyone inpatient diagnosis prior to the index date.

### 2.3 Study Design

ACHDs were identified from the Registry for Catastrophic Illness database, which is a sub-database of the NHIRD, between 2000 and 2010. Among ACHDs, the date of the first AF diagnosis (either before or after the CHD diagnosis) was considered the cohort entry date in the AF group. We further assigned the cohort entry date of ACHDs with AF to ACHDs without AF. This assignment approach is termed as “prescription time-distribution matching” which is known to deal with the immortal time bias [24]. We further matched the two groups at a 1:1 ratio based on sex, age group (18–54, 55–64, 65–74, and ≥75 years), and CHD type (ASD, VSD, TOF, and others). Furthermore, patients aged <18 years or with ischemic stroke (including transient ischemic attack) before the cohort entry date were excluded. Finally, patients in the AF group were matched to those in the non-AF group through propensity score matching at a 1:1 ratio. The variables included in the calculation of propensity scores were sex, age, ACHD type, ${\mathrm{CHA}}_{2}$${\mathrm{DS}}_{2}$-VASc score, obstructive sleep apnea (OSA), hypothyroidism, and the Charlson comorbidity index (CCI) score. The subsequent outcome comparisons were conducted on the matched cohort (**Supplementary Fig. 1**). Each patient was followed up from the index date to the date of event occurrence, death, or the end of database records (December 31, 2010), whichever occurred first.

### 2.4 Statistical Analysis

The SAS statistical package (version 9.4, SAS Institute Inc., Cary, NC, USA) was used for all statistical analyses. The risk of stroke events between the AF ACHD and non-AF ACHD groups was compared using the Cox proportional hazard model. Further, patients who underwent mechanical valve surgery are obligatory treated with warfarin lifelong, therefore a sensitivity analysis by excluding the patients who received mechanical valve surgery was conducted (the matching was re-performed).

Furthermore, subgroup analysis was performed based on the age group and CHD type. Finally, after excluding patients receiving antiplatelet therapy, we evaluated whether warfarin use was associated with ischemic stroke occurrence in ACHDs with AF. ACHDs with AF were subgrouped based on warfarin use and ${\mathrm{CHA}}_{2}$${\mathrm{DS}}_{2}$-VASc score (0–1 *vs.* ≥2). A *p* value of <0.05 was considered statistically significant.

## 3. Results

### 3.1 Atrial Fibrillation Prevalence and Overall Stroke Outcome

**Fig. 1.**
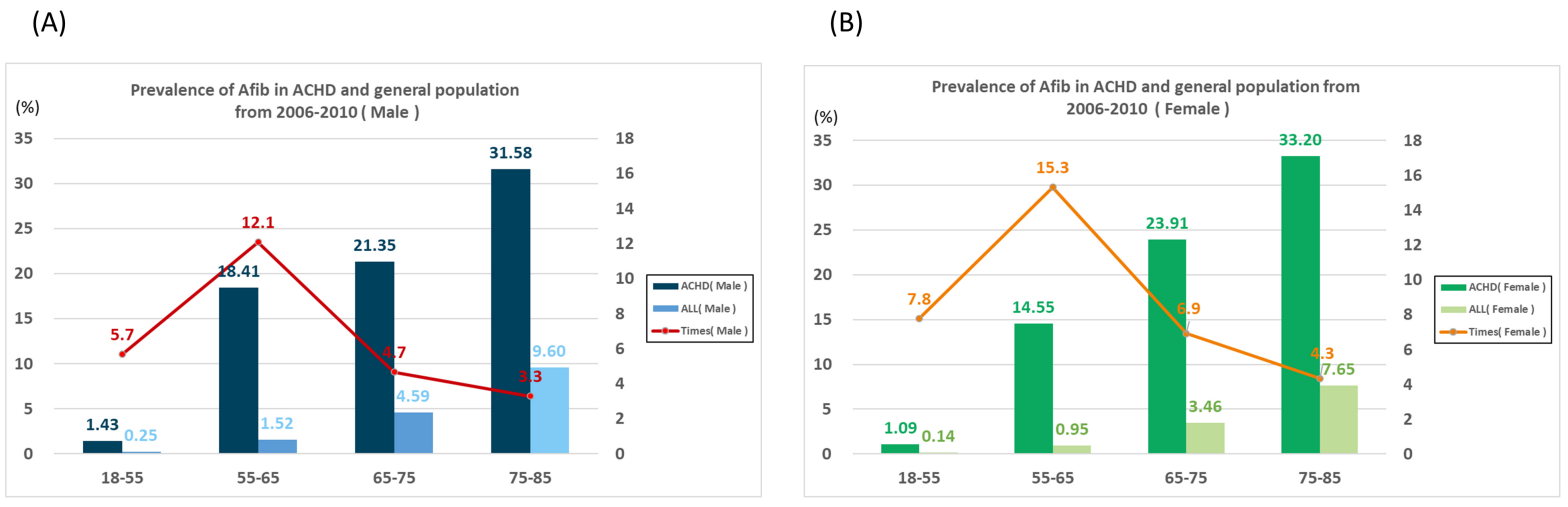
**Difference in the prevalence of atrial fibrillation (Afib) between the general population and adults with congenital heart diseases (ACHDs)**. (A) Male population. (B) Female population.

A total of 36,530 ACHDs were enrolled. Compared with the general population, ACHDs have an increased prevalence of AF in the 55–65 age group (up to 12.1 times in men and 15.3 times in women; Fig. 1).

**Fig. 2.**
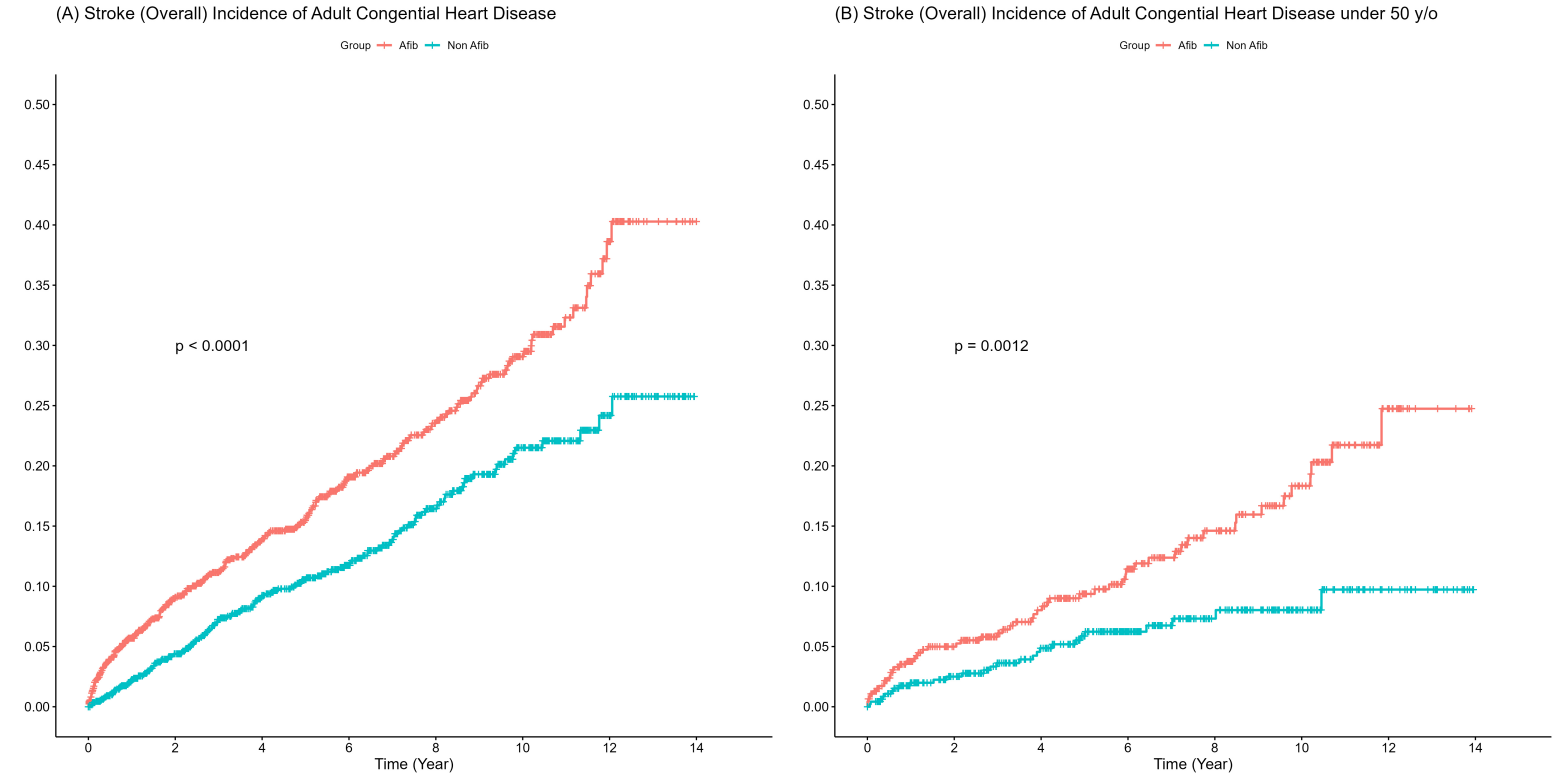
**Incidence of ischemic strokes in adults with congenital heart disease with and without atrial fibrillation (Afib) in the overall population and the population aged <50 years**. (A) Overall ischemic stroke incidence. (B) Ischemic stroke incidence in the population aged <50 years. y/o, years/old.

The baseline characteristics of ACHDs in the AF and non-AF groups after matching are presented in Table 1. The mean age was approximately 54 years, with no significant differences in age, sex, ${\mathrm{CHA}}_{2}$${\mathrm{DS}}_{2}$-VASc score , OSA, hypothyroidism, or CCI scores between the two groups. The risk of ischemic stroke events was higher in ACHDs with AF (adjusted hazard ratio [HR] = 1.45, 95% confidence interval [CI] = 1.11–1.92, *p* = 0.0093) than in those without AF (Table 2 and Fig. 2). Moreover, the analysis results remained consistent with the primary analysis even after excluding individuals who underwent mechanical valve surgery (adjusted hazard ratio [HR] = 1.82, 95% confidence interval [CI] = 1.31–2.54, *p* = 0.0004; Table 2).

**Table 1.** **Baseline characteristics of the AF and non-AF groups in ACHDs**.

Variables		AF ACHD (N = 1244)	Non-AF ACHD (N = 1244)	*p* value
Sex (%)	Female	686 (55.1)	659 (53.0)	0.296
	Male	558 (44.9)	585 (47.0)	
Age (%)	18~54	593 (47.7)	578 (46.5)	0.320
	55~64	275 (22.1)	313 (25.2)	
	65~74	267 (21.5)	255 (20.5)	
	Over 75	109 (8.8)	98 (7.9)	
Hypertension (%)	Yes	381 (30.6)	392 (31.5)	0.665
	No	863 (69.4)	852 (68.5)	
Diabetes mellitus (%)	Yes	153 (12.3)	154 (12.4)	1.000
	No	1091 (87.7)	1090 (87.6)	
Obstructive sleep apnea (%)	Yes	2 (0.2)	3 (0.2)	1.000
	No	1242 (99.8)	1241 (99.8)	
Hypothyroidism (%)	Yes	3 (0.2)	7 (0.6)	0.342
	No	1241 (99.8)	1237 (99.4)	
Congestive heart failure (%)	Yes	52 (4.2)	52 (4.2)	1.000
	No	1192 (95.8)	1192 (95.8)	
Prior stroke or TIA or thromboembolism (%)	No	1244 (100.0)	1244 (100.0)	NA
Vascular disease (%)	Yes	52 (4.2)	52 (4.2)	1.000
CCI score (mean (SD))		1.66 (2.19)	1.67 (2.04)	0.902
${\mathrm{CHA}}_{2}$${\mathrm{DS}}_{2}$-VASc score (mean (SD))		1.89 (1.73)	1.85 (1.67)	0.563
ACHD (%)	ASD	855 (68.7)	841 (67.6)	0.576
	VSD	437 (35.1)	411 (33.0)	0.290
	TOF	56 (4.5)	46 (3.7)	0.363
	Others	157 (12.6)	184 (14.8)	0.130
	≥2 types	415 (33.4)	421 (33.8)	0.832

ACHD, adults with congenital heart disease; AF, atrial fibrillation; ASD, atrial septal defect; CCI, Charlson comorbidity index; ${\mathrm{CHA}}_{2}$${\mathrm{DS}}_{2}$-VASc, congestive heart failure, hypertension, age ≥75 years, diabetes mellitus, stroke or transient ischemic attack, vascular disease, age 65–74 years, sex category; NA, not available; SD, standard deviation; TIA, transient ischemic attack; TOF, tetralogy of Fallot; VSD, ventricular septal defect; N, total number of participants.

**Table 2.** **Hazard ratios for incident ischemic stroke adjusted for age, sex, and CCI of the AF and non-AF groups in ACHDs**.

ACHD	AF ACHD (n/N)	Non-AF ACHD (n/N)	Adjusted HR (95% CI)	*p* value
Overall	129/1246	80/1246	1.45 (1.10, 1.92)	0.0093
Excluding mechanical valve replacement surgery	106/1055	52/1055	1.82 (1.31, 2.54)	0.0004
Age <50 years	35/478	18/509	1.46 (0.81, 2.63)	0.2071

ACHD, adults with congenital heart disease; Adjusted HR, adjusted hazard ratio for age and sex; AF, atrial fibrillation; CCI, Charlson comorbidity index; CI, confidence interval; n, number of participants with stroke; N, total number of participants.

In ACHDs aged <50 years, those with AF had a higher risk of ischemic stroke (adjusted HR = 1.46, 95% CI = 0.81–2.63, *p* = 0.2071) than those without AF (Table 2 and Fig. 2).

### 3.2 Ischemic Stroke Outcome Based on CHD Subtype

**Fig. 3.**
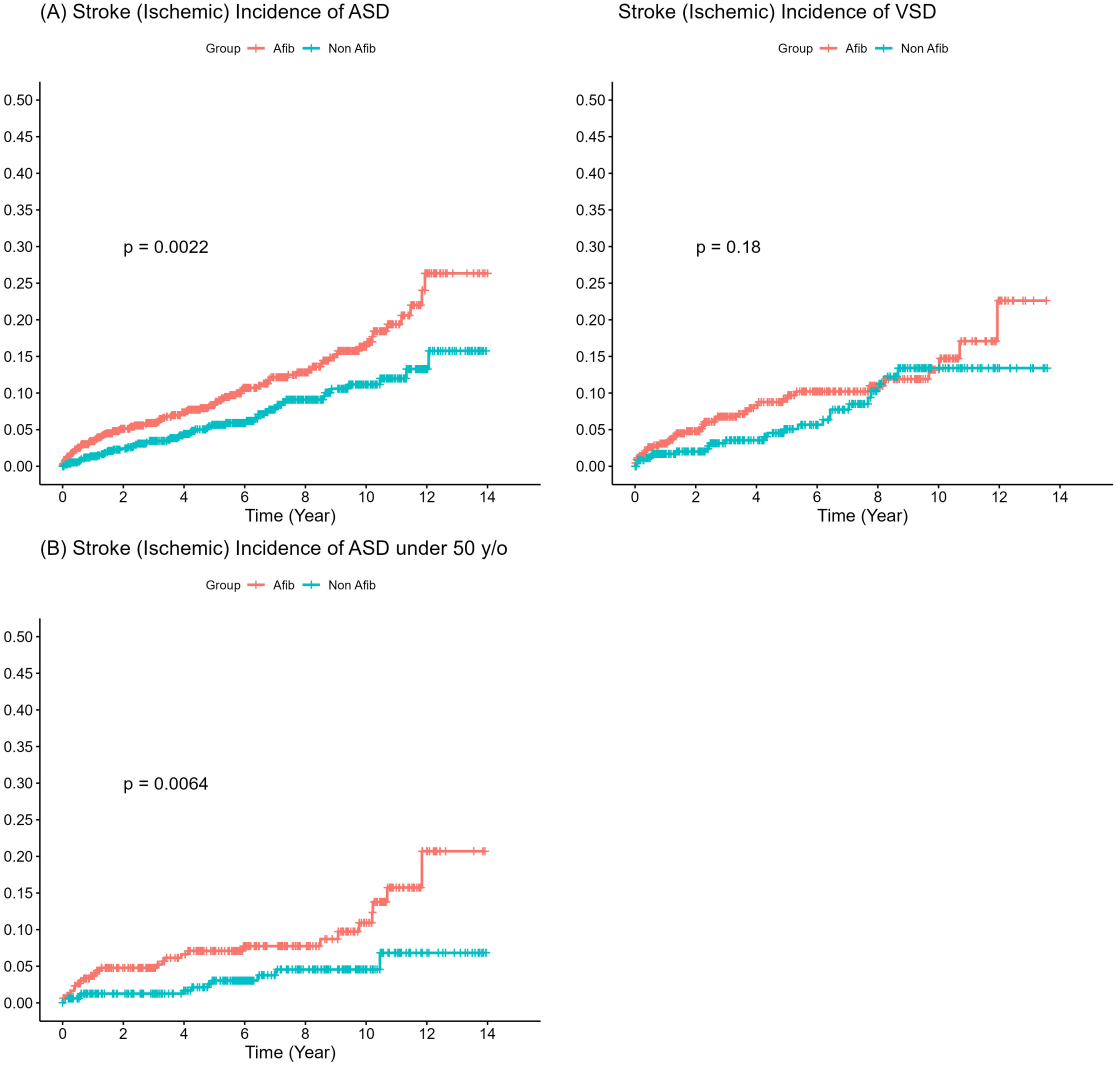
**Incidence of ischemic strokes in the population with atrial septal defect (ASD) and ventricular septal defect (VSD)**. (A) Overall ischemic stroke incidence (Left: ASD; Right: VSD). (B) ischemic stroke incidence in the ASD population aged <50 years. Afib, atrial fibrillation; y/o, years/old.

Among ACHDs with AF, the subgroups of ASD, VSD, TOF, and other CHDs demonstrated 1.57, 1.29, 2.29, and 1.66 times higher risk of ischemic stroke, respectively, compared to the corresponding subgroups in the non-AF group (Table 3, Fig. 3). Although the trend remains consistent, statistically significant ischemic stroke risk was observed only in the ASD subgroup with AF, even in individuals under the age of 50 (adjusted HR = 1.57, 95% CI = 1.11–2.21, *p* = 0.0097) (Table 3, Fig. 3).

**Table 3.** **Hazard ratios for incident ischemic stroke adjusted for age, sex, and CCI in the AF and non-AF groups of ACHD subtypes**.

Type of ACHD	AF ACHD (n/N)	non-AF ACHD (n/N)	Adjusted HR (95% CI)	*p* value
ASD	91/855	52/841	1.57 (1.11, 2.21)	0.0097
VSD	39/437	24/411	1.29 (0.77, 2.15)	0.3280
TOF	3/56	1/46	2.29 (0.20, 26.14)	0.5056
Others	22/157	15/184	1.66 (0.86, 3.22)	0.1296
≥2 types	48/415	27/421	1.62 (1.01, 2.60)	0.0457

ACHD, adults with congenital heart disease; Adjusted HR, adjusted hazard ratio for age and sex; AF, atrial fibrillation; ASD, ostium or secundum type atrial septal defect; CCI, Charlson comorbidity index; CI, confidence interval; n, the number of subjects with stroke; N, the total number of subjects; TOF, teratology of Fallot; VSD, ventricular septal defect.

### 3.3 Ischemic Stroke Outcomes of Diagnosis with Mixed CHD Subtype

Mixed type CHD is defined as the presence of two or more diagnosis CHD subtypes in a patient, and the ratio of this mixed type CHD is approximately 33% of the overall ACHD population (Table 1). Patients with mixed type CHD with AF had higher ischemic stroke risks than patients with mixed type CHD without AF (HR = 1.62, 95% CI = 1.01–2.6, *p* = 0.0457; Table 3).

### 3.4 Ischemic Stroke Risk according to ${\mathrm{CHA}}_{2}$${\mathrm{DS}}_{2}$-VASc Score in Patients with AF

After the exclusion of patients who underwent antiplatelet therapy (in the original cohort before propensity score matching), patients with AF were divided into two subgroups according to the ${\mathrm{CHA}}_{2}$${\mathrm{DS}}_{2}$-VASc score (0–1, ≥2, and overall). The baseline characteristics of warfarin therapy groups with lower and higher scores are listed (**Supplementary Table 1**). The annual incidence of ischemic stroke among those who use warfarin is presented in Table 4. Among those who do not use warfarin, the incidence of ischemic stroke among those with ${\mathrm{CHA}}_{2}$${\mathrm{DS}}_{2}$-VASc scores of 0–1 and ≥2 was 1.17% and 2.56%; among those who use warfarin, the incidence of stroke among those with ${\mathrm{CHA}}_{2}$${\mathrm{DS}}_{2}$-VASc scores 0–1 and ≥2 was 2.22% and 4.69%, respectively.

**Table 4.** Ischemic stroke outcomes in ACHDs with and without warfarin usage based on ${\mathrm{CHA}}_{2}$${\mathrm{DS}}_{2}$-VASc scoring*.

	ACHD without warfarin			ACHD with warfarin			Total		
CHA₂DS₂-VASc scoring	Number	Ischemic stroke incidence	Estimated year-risk %	Number	Ischemic stroke incidence	Estimated year-risk %	Number	Ischemic stroke incidence	Estimated year-risk %
0&1	460	32	1.17	189	24	2.22	649	56	1.47
2+	433	47	2.56	157	26	4.69	590	73	3.06
Overall	893	79	1.73	346	50	3.06	1239	129	2.08

ACHD, adults with congenital heart disease. ${\mathrm{CHA}}_{2}$${\mathrm{DS}}_{2}$-VASc, congestive heart failure, hypertension, age ≥75 years, diabetes mellitus, stroke or transient ischemic attack, vascular disease, age 65–74 years, sex category. *Patients with antiplatelet therapy usage were excluded from the analysis.

## 4. Discussion

### 4.1 Summary of Results

Our study showed that ACHDs (regardless of their sex or age) had a high prevalence of AF, particularly young women. However, the results of the AnTicoagulation and Risk Factors in Atrial Fibrillation (ATRIA) Study were different, wherein AF prevalence was 0.95% (95% CI, 0.94%–0.96%), and it was more common in men than in women (1.1% *vs.* 0.8%; *p* < 0.001) [25]. According to a Swedish database study of ACHDs, the risk of intra-atrial re-entrant tachycardia or AF is 22-fold higher in patients with CHD than in matched controls, with a prevalence of 8.3% in 42-year-olds [26]. In the current study, we found that AF prevalence was higher in ACHDs than in the general population in Taiwan. Furthermore, in ACHDs, the prevalence of AF increases with age and remains higher than that in the general population, with the highest prevalence observed in individuals under the age of 65. This may explain the increased stroke risk among young ACHDs in Taiwan.

Furthermore, among ACHDs, patients with AF have a higher ischemic stroke risk than those without AF. The Framingham study in 1991 demonstrated that AF is an independent risk factor for stroke in the general population [14]. Although the mechanism is unclear, ACHDs have some unique risk factors that are suspected to be associated with thromboembolic events, such as cyanosis, Fontan circulation, intracardiac shunt, and heart defect complexity [27]. Thus, thromboembolic risk in ACHDs might be underestimated. In the current study, among ACHDs, those with AF had nearly 1.5 times higher risk of ischemic stroke than those without AF. This trend was the same in the younger population (age <50 years).

Previous data on ACHDs from Asia and Europe showed that the overall stroke incidence was higher in ACHDs than in the general population, but details regarding stroke subtypes were limited [15, 28]. In this study, the overall ACHDs with AF as well as ASD, mixed type CHD subgroups showed a significantly increased risk of ischemic stroke. Importantly, in the young population (age <50 years), the trend was the same in the ASD group, which has not been reported in previous studies. Therefore, our study suggests that increased ischemic stroke incidence in ACHDs with AF even at young age, particularly ASD.

Previous attempts to use the ${\mathrm{CHA}}_{2}$${\mathrm{DS}}_{2}$-VASc score to predict thromboembolic risk in patients with CHD with AF have shown conflicting results [15]. Our study revealed that ACHDs with AF have a higher incidence of ischemic stroke, even if the ${\mathrm{CHA}}_{2}$${\mathrm{DS}}_{2}$-VASc score is low (<2). Traditionally, the ${\mathrm{CHA}}_{2}$${\mathrm{DS}}_{2}$-VASc score has been used to determine whether patients with AF can benefit from anticoagulation therapy. In 2014, experts recommended the use of the ${\mathrm{CHA}}_{2}$${\mathrm{DS}}_{2}$-VASc score to determine whether to use anticoagulation therapy in patients with CHD without prosthetic valves or significant valve disease [17]. However, in patients with CHD with prosthetic valves or significant valve disease and patients with moderate or severe CHD with intra-atrial re-entrant tachycardia or AF, using anticoagulants directly was recommended instead of ${\mathrm{CHA}}_{2}$${\mathrm{DS}}_{2}$-VASc score evaluation [17]. However, because ACHDs are few, sufficient comparative studies to evaluate the benefits and safety of anticoagulants are lacking. Few reports are available on the use of anticoagulant drugs in ACHDs with AF. Our study showed an annual risk of ischemic stroke incidence of approximately 1.47% and 3.06% at ${\mathrm{CHA}}_{2}$${\mathrm{DS}}_{2}$-VASc scores of <2 and >2, respectively. These results suggest that ACHDs with AF have a high risk of ischemic stroke, and that even those with a low ${\mathrm{CHA}}_{2}$${\mathrm{DS}}_{2}$-VASc score.

### 4.2 Limitations

This study has several limitations. First, information on many risk factors, such as smoking, obesity, metabolic syndrome, ventricular function, and severe valvular disease, was unavailable in the claims database, which may confound the results. Additionally, information on the International Normalized Ratio (INR) level and medical compliance of warfarin usage were unavailable in the database. Second, it is known that a patent foramen ovale (PFO) can increase the risk of ischemic stroke. However, the ICD-code based diagnosis cannot differentiate between ASD and PFO. To overcome this limitation and identify patients with ACHD in our study, we used the Registry for Catastrophic Illness database, a sub-dataset of the Taiwanese NHIRD that includes detailed clinical information and requires expert audited approval with formal medical echocardiography or cardiac catheterization reports. Using this rigorous criterion, we are confident that simple PFO cases were not classified as ACHD in our study. Third, detailed AF subtypes could not be validated from this cohort due to the limitations of the database. Moreover, information on the complexity and clinical functional status of CHD subtypes was limited. Fourth, it is important to acknowledge that this study has a retrospective observational design, which introduces the possibility of confounding factors influencing the outcomes of anticoagulation therapy. Despite the significantly high risk of ischemic stroke observed in ACHD patients with AF, as evidenced by the data presented in Table 4, it is likely that clinical and ethical biases have influenced the lack of protective effect observed with warfarin treatment. It should be noted that the decision to use warfarin in Taiwan is based on physician judgment, and this can be a challenging task given the higher propensity for bleeding and substantial fluctuations in therapeutic plasma levels among the Asian population. Close monitoring of anticoagulation therapy is crucial, especially in young populations [29]. Moreover, it is worth mentioning that the time spent within the therapeutic International Normalized Ratio (TTR) range is lower in Taiwan compared to other countries, even when considering data from randomized controlled trials such as RE-LY and ROCKET-AF [30, 31]. Furthermore, the baseline characteristics between the warfarin and non-warfarin groups were not balanced due to the limitation of small event numbers, which may have influenced the observed protective effect of warfarin. These findings raise questions about the potential benefits of anticoagulant therapy with warfarin in the ACHD population in Asia, specifically in Taiwan, and highlight the need for further prospective studies to address this issue. Additionally, in Taiwan, reimbursement policies do not allow the prescription of novel oral anticoagulants (NOACs) for stroke primary prevention in young adults with congenital heart disease (ACHDs) and atrial fibrillation. In 2020, a systematic review of the literature on NOAC use in ACHDs was conducted, and the results indicated that NOACs are safe and effective in ACHDs without mechanical prostheses [32]. Later, the international NOTE registry revealed that NOACs are safe and may be effective for thromboembolic prevention in adults with heterogeneous forms of congenital heart disease [33]. Further studies, such as the ongoing PROTECT AR (Apixaban in Adults With Congenital Heart Disease and Atrial Arrhythmias: the PROTECT-AR Study) trial, are necessary to confirm whether ACHDs with simple or complex diseases may benefit more from NOACs than from warfarin [34]. Finally, the study cohort sizes were small with few stroke events, but the number of ACHDs has been increasing in the past decade, and the management of clinically crucial issues such as AF and ischemic stroke in this population is important.

## 5. Conclusions

In summary, this study is the first to confirm the high prevalence of AF in Asian adults with congenital heart disease, which leads to an increased risk of ischemic stroke events. This risk is observed even at a young age (under 50 years old) or with a low ${\mathrm{CHA}}_{2}$${\mathrm{DS}}_{2}$-VASc score.

## Data Availability

The data underlying this article cannot be shared publicly due to ethical/privacy reasons. It could be applied followed Taiwan’s NHIRD policy.

## Supplementary Material

Supplementary material associated with this article can be found, in the online version, at https://doi.org/10.31083/j.rcm2408225.
