# Supplementary material for: Atrial Fibrillation in Adult Congenital Heart Increase Ischemic Stroke Risk Even at Low CHA2DS2-VASc Score
Source: Rev Cardiovasc Med. 2023 Aug 8;24(8):225. doi: 10.31083/j.rcm2408225 (PMC11266802; doi:10.31083/j.rcm2408225)
Supplement: Supplementary file 1 [file 2153-8174-24-8-225-s1.docx]

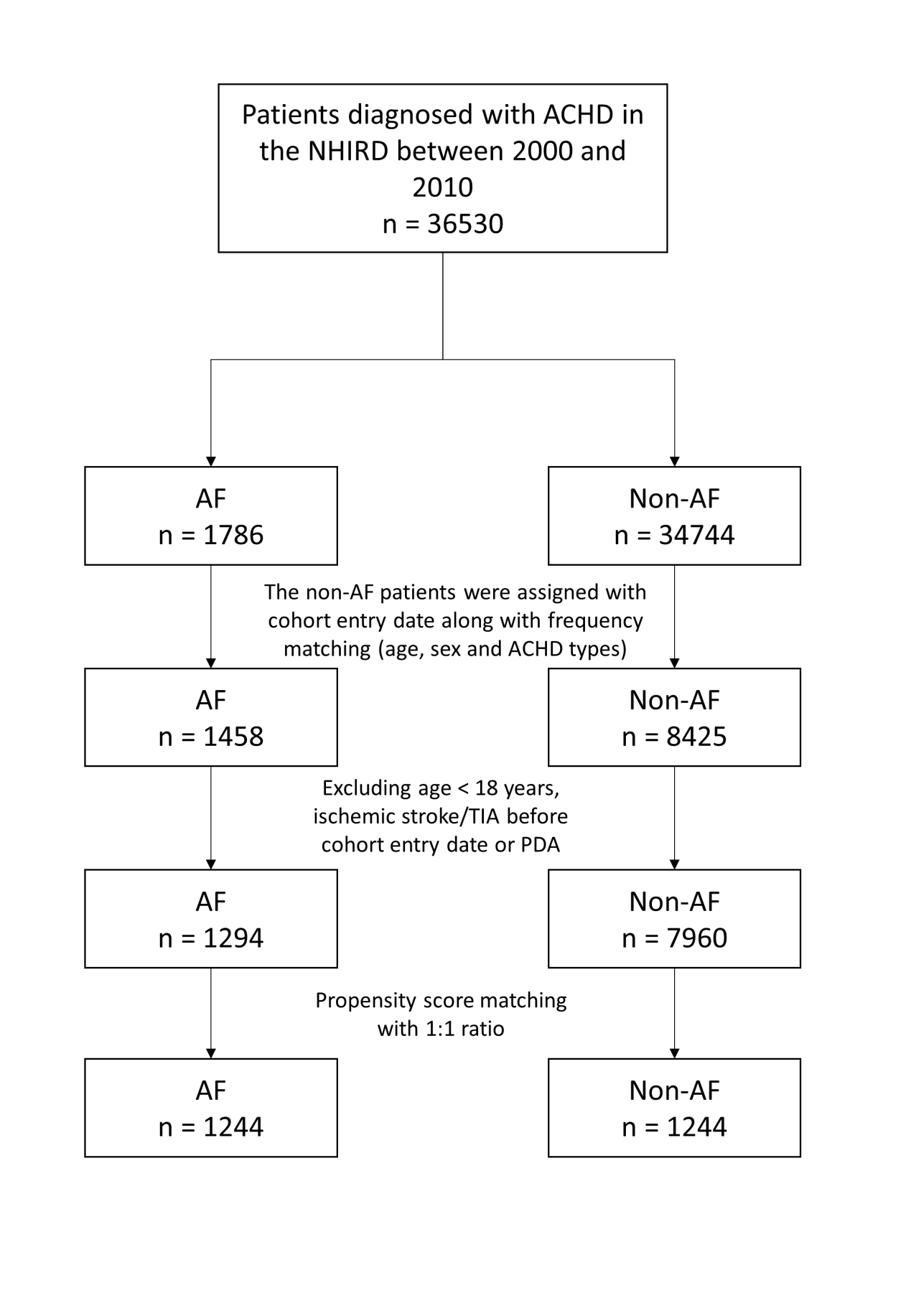


**Supplementary Fig. 1. Study flowchart.** ACHD = adult congenital heart disease; AF = atrial fibrillation; NHIRD = National Health Insurance Research Database; PDA (patent ductus ateriosus).

Supplementary Table 1. Baseline characteristics of the CHA₂DS₂-VASc score 0–1 and CHA₂DS₂-VASc score 2+ groups among ACHDs with AF who use warfarin.

| **Variables** |  | **CHA₂DS₂-VASc Score 0-1 group**  **(N = 189)** | **CHA₂DS₂-VASc Score 2+ groups**  **(N = 157)** | ***p*** |
| --- | --- | --- | --- | --- |
| SEX (%) | Female | 70 ( 37.0) | 111 ( 70.7) | <0.001 |
|  | Male | 119 ( 63.0) | 46 ( 29.3) |  |
| AGE (%) | 18~54 | 126 ( 66.7) | 48 ( 30.6) | <0.001 |
|  | 55~64 | 46 ( 24.3) | 45 ( 28.7) |  |
|  | 65~74 | 14 ( 7.4) | 49 ( 31.2) |  |
|  | Over 75 | 3 ( 1.6) | 15 ( 9.6) |  |
| Hypertension (%) | Yes | 0 ( 0.0) | 97 ( 61.8) | <0.001 |
|  | No | 189 (100.0) | 60 ( 38.2) |  |
| Diabetes mellitus (%) | Yes | 4 ( 2.1) | 41 ( 26.1) | <0.001 |
|  | No | 185 ( 97.9) | 116 ( 73.9) |  |
| Obstructive sleep apnea (%) | Yes | 1 ( 0.5) | 0 ( 0.0) | 1.000 |
|  | No | 188 ( 99.5) | 157 (100.0) |  |
| Hypothyroidism (%) | Yes | 0 ( 0.0) | 0 ( 0.0) | NA |
|  | No | 189 (100.0) | 157 (100.0) |  |
| Congestive heart failure(%) | Yes | 36 ( 19.0) | 96 ( 61.1) | <0.001 |
|  | No | 153 ( 81.0) | 61 ( 38.9) |  |
| Prior stroke or TIA or thromboembolism (%) | Yes | 0 ( 0.0) | 0 ( 0.0) | NA |
|  | No | 189 (100.0) | 157 (100.0) | NA |
| Vascular disease(%) | Yes | 1 ( 0.5) | 13 ( 8.3) | 0.001 |
|  | No | 188 ( 99.5) | 144 ( 91.7) |  |
| CCI Score (mean (SD)) |  | 0.79 (1.24) | 2.76 (2.21) | <0.001 |
| **CHA₂DS₂-VASc** Score (mean (SD)) |  | 0.59 (0.49) | 3.27 (1.32) | <0.001 |

ACHD = adults with congenital heart disease; AF = atrial fibrillation; CHA₂DS₂-VASc = congestive heart failure, hypertension, age ≥75 years, diabetes mellitus, stroke or transient ischemic attack, vascular disease, age 65–74 years, sex category; CCI = Charlson comorbidity index; SD = standard deviation; TIA = transient ischemic attack.
